# What Traits Should Be Measured for Biomass in Kenaf?

**DOI:** 10.3390/plants10071394

**Published:** 2021-07-07

**Authors:** Jaeyoung Kim, Gyung Deok Han, Gopi Muthukathan, Renato Rodrogues, Do Yoon Hyun, Seong-Hoon Kim, Ju-Kyung Yu, Jieun Park, Soo-Cheul Yoo, Yong Suk Chung

**Affiliations:** 1 Department of Plant Resources and Environment, Jeju National University, Jeju 63243, Korea; baron7798@jejunu.ac.kr (J.K.); hangds@hanmail.net (G.D.H.); pje0195@naver.com (J.P.); 2 ICAR-National Research Centre for Banana, Tiruchirappalli 620102, India; gopimusa@gmail.com; 3 Institute of Mathematics and Statistics, Federal University of Goias, Goiania 74001, Brazil; renato.rrsilva@ufg.br; 4 National Agrobiodiversity Center, National Institute of Agricultural Sciences (NAS), RDA, Jeonju 54875, Korea; dyhyun@korea.kr (D.Y.H.); shkim0819@korea.kr (S.-H.K.); 5 Seeds Research, Syngenta Crop Protection LLC, Research Triangle Park, NC 27709, USA; yjk0830@hotmail.com; 6 Department of Plant Life and Environmental Science, Hankyong National University, Anseong-si 17579, Korea

**Keywords:** kenaf, germplasm, breeding, key traits, industrial crop

## Abstract

Kenaf (*Hibiscus cannabinus* L.) is widely used as an important industrial crop. It has the potential to act as a sustainable energy provider in the future, and contains beneficial compounds for medical and therapeutic use. However, there are no clear breeding strategies to increase its biomass or leaf volume. Thus, to attain an increase in these parameters, we examined potential key traits such as stem diameter, plant height, and number of nodes to determine the relationship among them. We hypothesized that it would be easier to reduce the amount of time and labor required for breeding if correlations among these parameters are identified. In this study, we found a strong positive correlation between height and number of nodes (Spearman’s Rho = 0.67, *p* < 0.001) and number of nodes and stem diameter (Spearman’s Rho = 0.65, *p* < 0.001), but a relatively low correlation (Spearman’s Rho = 0.34, *p* < 0.01) between height and stem diameter in the later stages of kenaf growth. We suggest that an efficient breeding strategy could be devised according to the breeding purpose, considering the correlations between various individual traits of kenaf.

## 1. Introduction

Kenaf (*Hibiscus cannabinus* L.) is an important industrial crop worldwide [1]. It is cultivated in more than 20 countries because of its importance and various roles in industrial and agricultural applications; it is a constituent of paper and pulp, fabrics, textiles, biocomposites, insulation mats, absorption materials, animal bedding, medicinal formulations, musical instruments, and value-added plant-based foods [2,3,4,5,6]. These numerous applications are due to kenaf’s fibrous stems and functional compounds. It is characterized by rapid growth, with an average increase of 10 cm in a single day, and a large biomass, reaching 4–6 m in height [7,8]. These plants also have a wide adaptability in various climates and soils [9]. Consequently, its cultivars have spread to Asia through Southern and Western Africa, although its origin might have been Zambia or the surrounding areas [10].

Importantly, owing to its large volume of biomass, kenaf could be a potential material for sustainable energy supply in the future, and its useful phytocompounds and phytol from leaves could be extracted for medical purposes [11]. Kenaf leaf extract contains many plant compounds, including phytol and linolenic acid, which are known to have various health benefits [12,13,14]. Its leaves have been used to treat dysentery, blood and throat disorders, and in the management of atherosclerosis [15,16]. A recent study showed that the fortification of bread using kenaf leaves improved the total dietary fiber content of the former [17]. Furthermore, leaves contribute to an increase in total biomass. After drying, kenaf leaf biomass is approximately 40% of its total biomass [18,19].

Despite the importance of kenaf leaves for total biomass increment and other uses, kenaf fibers have been relatively more useful for industrial applications. Therefore, increasing the fiber production of kenaf is a primary breeding goal [18,19]. Most kenaf breeding programs in the United States are aimed at developing varieties suitable for the production of fibers, while in Cuba, Guatemala, and a few states such as Florida, they are aimed at producing high-yielding and disease-resistant varieties [20,21,22]. Fiber yield, which is related to biomass in kenaf, is strongly associated with bark thickness, stem diameter, and plant height [20,21]. However, research on the correlations of traits that affect biomass or traits related to biomass is insufficient. Information on the correlation of key traits, such as stem diameter, leaves by number of nodes, and height for kenaf breeding, is lacking, making its breeding inefficient. In other plants, these key traits have been reported to be related to biomass. For instance, in rice, the height of a plant is used to estimate the biomass [23]. In sorghum, a thicker stem diameter is preferred as this indicates a greater biomass yield [24]. In addition, Mauro-Herrera and Doust [25] have suggested that biomass is highly correlated with the height of the plant and the number of nodes on the main stem. Furthermore, in the giant reed, a higher number of nodes on the stem means a higher amount of meristem tissue, and thereby, a larger amount of biomass [26].

In this study, the growth patterns of the potential key traits mentioned above were examined over time in 23 kenaf cultivars, and the correlation between each trait was determined. By elucidating the correlation among the traits studied, biomass increment-related breeding in kenaf could be established. Furthermore, we measured the traits mentioned above at different growth stages to determine whether early selection for each trait is possible.

## 2. Materials and Methods

### 2.1. Experiment Site and Plant Materials

The experiment was conducted from 2 May 2019 to 5 September 2019 in the Jeju National University Test Field, Korea (33°27′35.7″ N 126°33′50.3″ E DMS). The average temperature ranged from 16.6 °C to 30.6 °C, and the total precipitation was measured to be 1056.7 mm during the experiment (Table 1). Kenaf cultivars were provided by the Rural Development Administration (RDA, Korea) and SJ Global Co., Ltd. (https://koreakenaf.modoo.at/, accessed on 6 June 2021, Bucheon, Korea) (Table 2).

On 2 May 2019, 15 individuals of each of the 24 cultivars were planted in a row at a distance of 25 cm between each other in one planting section. A total of 24 plots of each cultivar were replicated three times and randomly arranged. The distance between each section was 50 cm, and the distance between each row was approximately 100 cm. All individuals were well irrigated from 14 days after planting, once a day, until the end of the experiment. Additionally, only data from 23 cultivars were used in the experiment because of the lack of germination in EF-2 and lodged individuals due to the influence of typhoons during their growth, meaning that they had to be supported with stakes.

### 2.2. Measurements

The number of nodes, stem diameter, and height of three randomly selected kenaf individuals from each section were measured in four sets on days 75 (15 June 2019), 86 (26 July 2019), 103 (12 October 2019), and 127 (5 September 2019) after planting. The number of nodes was measured by counting the nodes of the main stem as these were visible to the naked eye. The stem diameter was estimated at the middle of the first and second nodes of the main stem using a Vernier caliper, and the height was measured from the ground to the tip of the individuals using a measuring tape.

### 2.3. Statistical Analysis

Data analysis was performed using R software (Ver. 1.3.1056., RStudio Team, R Foundation for Statistical Computing, Boston, MA, USA). Non-parametric tests (Kruskal–Wallis test, post hoc Dunn’s test with Benjamini–Hochberg FDR correction) were applied to compare the stem diameter, number of nodes, and height of the 23 kenaf cultivars. Spearman’s rank correlations were used to determine the degree of agreement of the ranking of each parameter.

## 3. Results and Discussion

**Figure 1:**
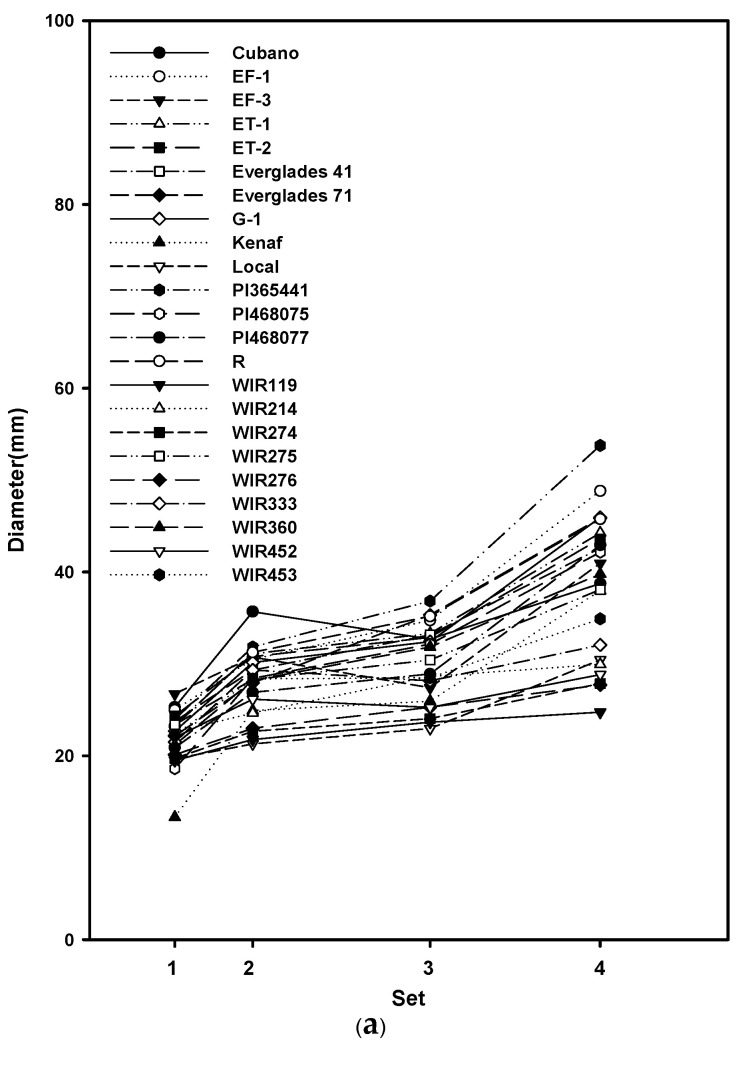

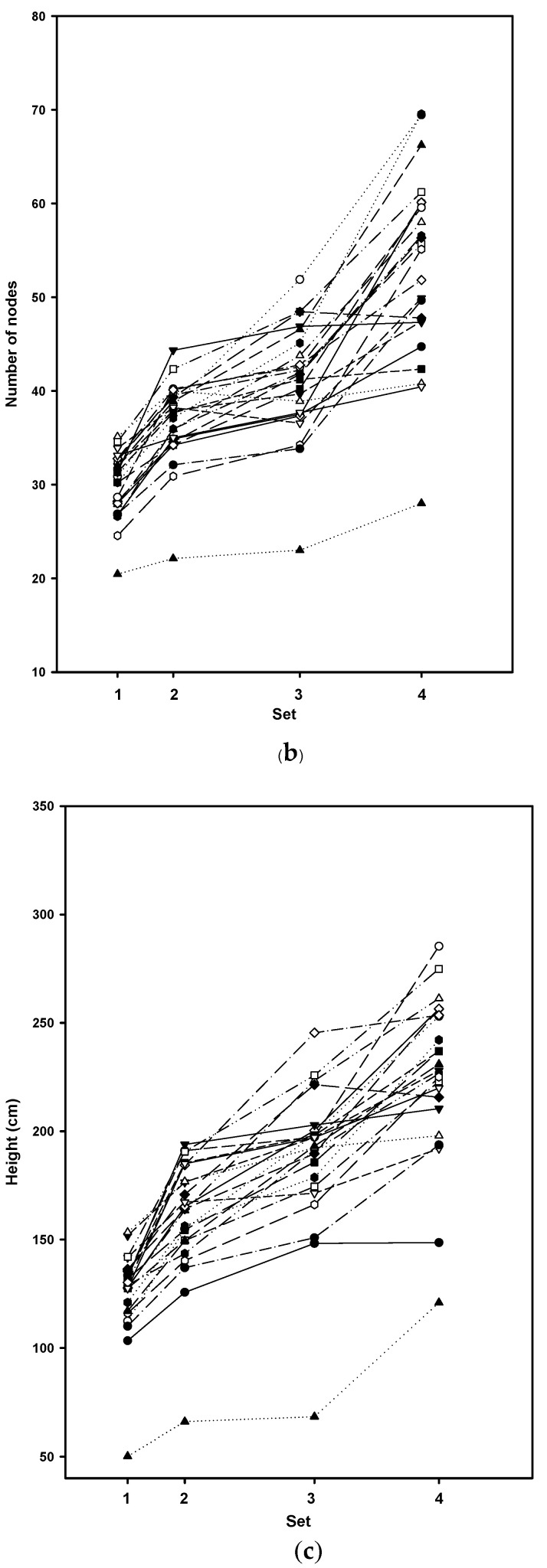
(**a**) Stem diameter (mm); (**b**) Number of nodes; and (**c**) Height (cm) of 23 cultivars at four different growth stages.

Significant differences in the germplasms in terms of the stem, nodes, and shoot tip were found, except in the stem and nodes of plants in Set 2 (Table 3). The lack of differences in all replications implies that the data were consistent and reproducible. However, the rank of each trait did not remain the same (Figure 1, Table 4, Table 5 and Table 6). Although the rank of each trait was similar in the majority of germplasms, some of them decreased or increased dramatically. This strongly indicates that the selection must be performed at the end of the growth stage. In addition, differences in the germplasms of different tissues at different time points suggest that the growth rate of each germplasm is different in different environments, which could be worth examining.

We found that correlations among traits varied at different growth stages (Table 7). This could be due to the rank changes mentioned above, meaning that the growth rates for each trait in each germplasm are diverse. Assuming selection would be made at the end of the growth stage, the correlation between the number of nodes and stem diameter, as well as that between the number of nodes and height, were relatively high at 0.65 and 0.67, respectively. This could be because the number of nodes increases both horizontally and vertically as plant diameter and plant height, respectively, increase. Hence, an increase in the number of leaves is a direct function of stem diameter and plant height, which are much easier to measure for efficient plant selection for breeding purposes. With the same assumption, height and stem diameter had a low correlation (0.34). This indicates that they need to be measured separately to increase biomass because biomass is highly associated not only with height but also with stem diameter.

The high correlation can be attributed to two possibilities—co-selection and genetic linkage—while the reasons are the opposite for a low correlation. Plant height is the result of primary growth, and its diameter is that of secondary growth [27]. The question to be considered is how the two are related. In rice, there was no overlap between quantitative trait loci (QTLs) for increased stem diameter and QTLs for plant height [28]. In addition, in soybean, many QTLs for height and the number of nodes are not linked to each other [29]. Likewise, in Eucalyptus, woody plants, Chinese silver grass, and herbal plants, height and circumference have a strong phenotypic correlation, although many QTLs for height and circumference have not been linked to each other [30]. In addition, the chance of co-selection is low, considering that the plant materials used in the current study are mostly germplasm.

In summary, both stem diameter and height should be measured for a more effective biomass-based breeding strategy. In addition, to breed a kenaf cultivar with many leaves (for obtaining the functional compounds or for other purposes), height or stem diameter could be measured because they have a high correlation with the number of nodes. Additionally, height or stem diameter are more accessible and measurable traits, especially height, and could be estimated using an unmanned aerial vehicle for easier selection [31]. Moreover, selection should be made at the end of the growth stage because the rank of each trait varies significantly in this phase of growth.

## 4. Conclusions

In this study, correlations and growth patterns of major traits, such as stem diameter, number of nodes, and height over time, were confirmed in various germplasms. Since different germplasms have different traits, it is necessary to screen them according to the breeding purposes. In addition, this study showed a strong correlation between the number of nodes and height over time and a weak correlation between stem diameter and height. We showed that the correlation of each trait in kenaf implies that the breeding strategy could be made more efficient if this information is utilized.

## Figures and Tables

**Table 1:** Climate variables of experiment site, from May to September 2019.

	May	June	July	October	September ^1^
Average min Temperature (°C)	16.6	19.0	23.2	25.5	22.4
Average max Temperature (°C)	23.8	25.1	27.9	30.6	27.6
Total monthly precipitation (mm)	42.8	145.8	510.1	242.3	115.7

^1^ From 1–5 September.

**Table 2:** Twenty-four kenaf (*Hibiscus cannabinus* L.) cultivars were tested in the experiment.

Entry	Origins
Cubano	Cuba
Everglades 41	US
Everglades 41	US
Kenaf	Myanmar
Local	Africa
PI365441	Taiwan
PI468075	US
PI468077	US
WIR119	India
WIR214	Iran
WIR274	Iran
WIR275	Iran
WIR276	Iran
WIR333	France
WIR360	Italy
WIR452	China
WIR453	Iran
EF-1	-
EF-2	-
EF-3	-
ET-1	-
ET-2	-
G-1	-

**Table 3:** Kruskal–Wallis rank sum test at four growth stages.

		Stem Diameter				Number of Nodes				Height			
Source	Df	Set 1 ^1^	Set 2	Set 3	Set 4	Set 1	Set 2	Set 3	Set 4	Set 1	Set 2	Set 3	Set 4
Replication	2	NS ^2^	NS	NS	NS	NS	NS	NS	NS	NS	NS	NS	NS
Entries	22	* ^3^	NS	**	**	**	NS	*	**	*	**	**	***

^1^ Set 1, Set 2, Set 3, and Set 4 measured on 15 July, 26 July, 12 August, and 5 September. ^2^ NS, nonsignificant at *p* > 0.05. ^3^ * Significant at the 0.05, ** Significant at the 0.01, and *** Significant at the 0.001 probability level.

**Table 4:** Variation among Kenaf (*Hibiscus cannabinus* L.) cultivar in stem diameter at four growth stages.

Set 1 ^1^		Set 2		Set 3		Set 4	
Cultivar	Diameter ^2^	Cultivar	Diameter	Cultivar	Diameter	Cultivar	Diameter
EF-3	26.74 ± 0.92 a ^3^	Cubano	35.70 ± 7.22 a	PI365441	36.84 ± 3.24 a	PI365441	53.77 ± 2.46 a
Cubano	25.29 ± 2.73 ab	PI365441	31.84 ± 1.70 a	Everglades 71	35.32 ± 1.65 a	EF-1	48.83 ± 3.77 ab
EF-1	24.98 ± 2.36 ab	R	31.26 ± 1.57 a	R	35.18 ± 2.88 a	G-1	45.94 ± 3.10 ab
ET-2	24.35 ± 0.06 ab	ET-1	31.09 ± 3.05 a	EF-1	34.73 ± 3.73 ab	Everglades 71	45.86 ± 0.69 ab
PI365441	23.99 ± 0.95 ab	ET-2	30.80 ± 1.12 a	ET-1	33.31 ± 3.52 ab	R	45.75 ± 3.04 ab
Everglades 41	23.75 ± 0.82 ab	EF-3	30.74 ± 2.88 a	Everglades 41	33.15 ± 2.05 ab	ET-1	44.26 ± 2.14 abc
ET-1	23.43 ± 2.47 ab	EF-1	30.51 ± 3.40 a	ET-2	32.89 ± 2.94 ab	ET-2	43.6 ± 1.30 abc
WIR275	23.39 ± 1.88 ab	G-1	30.16 ± 3.59 a	Cubano	32.72 ± 7.61 abcd	PI468077	42.93 ± 1.75 ab
R	23.24 ± 1.56 ab	WIR333	29.35 ± 1.06 a	G-1	32.42 ± 2.16 abc	Everglades 41	42.61 ± 10.11 abcd
G-1	22.70 ± 2.93 ab	Everglades 41	29.32 ± 0.50 a	PI468075	32.15 ± 4.21 abcd	PI468075	42.16 ± 6.59 abcd
WIR214	22.60 ± 0.28 ab	PI468075	28.43 ± 2.92 a	WIR360	31.84 ± 3.02 abcd	EF-3	40.95 ± 2.01 abcde
WIR453	22.26 ± 3.01 ab	WIR453	28.36 ± 1.69 a	WIR275	30.42 ± 2.06 abcd	WIR360	39.77 ± 4.29 abcde
Everglades 71	22.18 ± 1.21 ab	WIR275	28.31 ± 2.79 a	PI468077	28.90 ± 2.70 abcd	Cubano	38.74 ± 2.44 bcdef
WIR452	21.98 ± 0.86 ab	WIR360	28.19 ± 2.52 a	WIR214	28.65 ± 1.59 abcd	WIR275	38.07 ± 2.57 bcdef
WIR333	21.50 ± 0.83 ab	Everglades 71	27.95 ± 0.23 a	WIR453	28.49 ± 0.46 abcd	Kenaf	37.98 ± 2.38 bcdef
WIR360	21.18 ± 0.87 ab	PI468077	26.90 ± 2.85 a	WIR333	28.15 ± 1.21 abcd	WIR453	34.91 ± 3.59 bcdef
PI468077	20.89 ± 1.82 ab	WIR452	26.15 ± 0.89 a	EF-3	27.41 ± 0.99 abcd	WIR333	32.05 ± 3.63 cdef
WIR276	20.12 ± 0.43 ab	Kenaf	24.93 ± 2.03 a	Kenaf	25.94 ± 1.35 bcd	Local	30.45 ± 6.09 cdef
WIR274	19.72 ± 0.74 ab	WIR214	24.66 ± 0.49 a	WIR276	25.29 ± 1.74 bcd	WIR214	29.95 ± 1.65 def
Local	19.66 ± 1.38 ab	WIR276	23.00 ± 0.82 a	WIR452	25.27 ± 0.48 bcd	WIR452	28.84 ± 2.60 def
WIR119	19.49 ± 1.40 ab	WIR274	22.66 ± 3.41 a	WIR274	24.05 ± 1.18 cd	WIR274	27.87 ± 1.79 ef
PI468075	18.59 ± 2.73 ab	WIR119	21.78 ± 0.77 a	WIR119	23.66 ± 0.24 d	WIR276	27.72 ± 1.58 ef
Kenaf	13.31 ± 0.15 b	Local	21.30 ± 2.42 a	Local	22.97 ± 1.89 d	WIR119	24.76 ± 0.56 f

^1^ Set 1, Set 2, Set 3, and Set 4 measured on 15 July, 26 July, 12 August, and 5 September. ^2^ Unit = mm. ^3^ Means of ± standard errors followed by different letters within columns are significantly different by Dunn’s test with Benjamini–Hochberg. Non-parametric rank data were used for statistical analysis; however, untransformed data are presented.

**Table 5:** Variation among Kenaf (*Hibiscus cannabinus* L.) cultivar in the number of nodes at four growth stages.

Set 1 ^1^		Set 2		Set 3		Set 4	
Cultivar	Number of Nodes	Cultivar	Number of Nodes	Cultivar	Number of Nodes	Cultivar	Number of Nodes
WIR214	35.11 ± 1.44 a ^2^	WIR119	44.33 ± 2.33 a	EF-1	51.89 ± 8.22 ab	WIR453	69.56 ± 8.66 ab
WIR275	34.56 ± 2.56 ab	WIR275	42.33 ± 2.91 ab	WIR275	48.44 ± 2.38 a	EF-1	69.44 ± 2.44 a
Local	33.89 ± 1.64 a	R	40.22 ± 2.90 ab	WIR276	48.44 ± 3.26 ab	WIR360	66.22 ± 10.39 abcd
WIR452	33.11 ± 0.87 abc	WIR333	40.11 ± 1.72 ab	WIR119	46.89 ± 2.44 ab	WIR275	61.22 ± 3.58 abc
WIR276	32.78 ± 0.80 abcd	WIR214	40.00 ± 2.46 ab	WIR360	46.56 ± 2.89 ab	G-1	60.11 ± 0.48 abcd
WIR333	32.78 ± 0.68 abcd	Everglades 41	39.56 ± 1.68 ab	WIR453	45.11 ± 1.87 ab	ET-2	60.00 ± 5.35 abcde
Everglades 41	32.56 ± 2.00 abcde	WIR276	39.33 ± 1.02 ab	ET-1	43.78 ± 3.32 abc	R	59.56 ± 4.12 abcdef
EF-3	32.00 ± 1.17 abcdef	WIR360	38.89 ± 1.47 ab	WIR333	42.78 ± 1.06 abc	ET-1	58.00 ± 3.47 abcdef
WIR360	32.00 ± 2.04 abcdef	EF-1	38.44 ± 2.45 ab	R	42.56 ± 7.67 abc	PI365441	56.56 ± 2.51 abcdefg
WIR119	31.33 ± 0.69 abcdefg	Local	38.22 ± 1.87 ab	Everglades 41	42.22 ± 3.76 abc	Everglades 71	56.33 ± 2.04 abcdefg
WIR274	31.33 ± 1.02 abcdefg	EF-3	37.89 ± 2.79 ab	PI365441	41.94 ± 3.58 abc	Everglades 41	55.67 ± 7.37 abcdefgh
EF-1	30.89 ± 0.91 abcdefg	WIR274	37.67 ± 0.38 ab	Everglades 71	41.78 ± 1.47 abc	PI468075	55.11 ± 4.41 abcdefghi
ET-2	30.22 ± 0.56 abcdefgh	WIR453	37.11 ± 3.95 ab	WIR274	41.22 ± 1.64 abc	WIR333	51.83 ± 3.59 bcdefghij
WIR453	30.22 ± 1.82 abcdefg	PI365441	35.94 ± 1.00 ab	ET-2	40.22 ± 1.28 abc	Local	49.89 ± 9.92 cdefghijk
R	28.67 ± 1.84 bcdefgh	ET-1	35.89 ± 1.60 ab	EF-3	39.67 ± 0.19 abc	PI468077	49.67 ± 0.84 cdefghijk
Everglades 71	28.22 ± 1.28 defgh	WIR452	35.00 ± 1.84 ab	WIR214	38.89 ± 4.08 abc	WIR276	47.78 ± 2.70 defghijk
G-1	28.00 ± 2.14 cdefgh	Cubano	34.89 ± 4.83 ab	WIR452	37.67 ± 1.20 abc	EF-3	47.42 ± 1.11 fghijk
ET-1	27.89 ± 0.78 efgh	Everglades 71	34.56 ± 0.91 ab	Cubano	37.50 ± 6.06 abc	WIR119	47.33 ± 2.22 efghijk
PI468077	26.89 ± 1.68 fgh	ET-2	34.22 ± 1.75 ab	G-1	37.33 ± 3.48 abc	Cubano	44.72 ± 3.82 ghijk
Cubano	26.78 ± 1.89 fgh	G-1	34.22 ± 2.06 ab	Local	36.56 ± 2.22 abc	WIR274	42.33 ± 4.58 hijk
PI365441	26.61 ± 1.11 gh	PI468077	32.11 ± 4.89 ab	PI468075	34.22 ± 3.27 abc	WIR214	40.78 ± 2.31 jk
PI468075	24.56 ± 3.09 gh	PI468075	30.89 ± 3.04 ab	PI468077	33.83 ± 2.35 bc	WIR452	40.44 ± 4.58 ijk
Kenaf	20.44 ± 0.59 h	Kenaf	22.11 ± 1.06 b	Kenaf	23.00 ± 1.20 c	Kenaf	28.00 ± 2.52 k

^1^ Set 1, Set 2, Set 3, and Set 4 measured on 15 July, 26 July, 12 August, and 5 September. ^2^ Means of ± standard errors followed by different letters within columns are significantly different by Dunn’s test with Benjamini–Hochberg. Non-parametric rank data were used for statistical analysis; however, untransformed data are presented.

**Table 6:** Variation among Kenaf (*Hibiscus cannabinus* L.) cultivar in height at four growth stages.

Set 1 ^1^		Set 2		Set 3		Set 4	
Cultivar	Height ^2^	Cultivar	Height	Cultivar	Height	Cultivar	Height
WIR214	153.33 ± 7.75 a ^3^	WIR119	193.89 ± 4.72 a	WIR333	245.44 ± 4.07 a	R	285.33 ± 24.27 ab
EF-3	151.78 ± 4.29 a	R	191.00 ± 12.10 abc	WIR275	225.78 ± 12.26 ab	WIR275	274.89 ± 8.57 a
WIR275	142.00 ± 8.34 ab	WIR275	190.56 ± 10.20 ab	ET-1	223.33 ± 25.29 abc	ET-1	261.22 ± 22.02 abc
Local	141.67 ± 5.06 ab	WIR274	185.33 ± 1.84 abc	WIR276	221.44 ± 10.23 ab	Everglades 71	256.44 ± 16.25 abcd
Everglades 71	136.33 ± 6.94 abc	WIR452	185 ± 4.10 abc	WIR119	202.89 ± 10.79 abcd	G-1	256.44 ± 2.95 abc
WIR276	135.44 ± 2.98 abc	WIR333	184.39 ± 8.05 abc	G-1	200.33 ± 3.51 abcd	WIR333	253.50 ± 17.15 abcd
R	133.44 ± 16.48 abc	WIR214	176.78 ± 14.35 abcde	EF-1	198.44 ± 17.12 abcd	EF-1	253.00 ± 18.38 abcd
WIR274	133.22 ± 13.64 abc	EF-3	176.44 ± 8.73 abcd	WIR274	198.00 ± 8.14 abcd	WIR453	242.11 ± 13.57 abcde
WIR119	132.22 ± 3.35 abc	WIR276	170.78 ± 8.83 abcde	R	197.22 ± 33.21 abcd	ET-2	237.00 ± 1.54 abcde
ET-2	132.00 ± 0.51 abc	Local	167.22 ± 7.95 abcde	EF-3	197.17 ± 7.41 abcd	WIR274	236.92 ± 12.43 abcde
WIR333	130.22 ± 7.75 abc	G-1	165.22 ± 2.63 abcde	WIR452	197.11 ± 9.56 abcd	WIR360	230.89 ± 10.37 abcdef
PI365441	128.94 ± 8.04 abc	Everglades 71	164.11 ± 7.53 abcde	WIR360	193.11 ± 10.29 abcde	EF-3	227.92 ± 2.55 bcdefg
ET-1	128.11 ± 6.27 abc	ET-1	163.44 ± 9.34 abcde	WIR214	192.33 ± 8.21 abcde	PI365441	226.56 ± 9.72 cdefg
G-1	127.67 ± 6.89 abc	WIR453	156.22 ± 6.88 cdef	PI365441	191.06 ± 12.35 abcde	PI468075	225.00 ± 20.34 cdefg
WIR452	127.44 ± 18.93 abc	EF-1	154.89 ± 16.23 bcdef	Everglades 71	189.67 ± 11.18 abcde	Everglades 41	223.00 ± 5.59 cdefgh
Everglades 41	127.44 ± 6.79 abc	ET-2	154.44 ± 4.90 def	ET-2	185.67 ± 5.03 abcde	WIR452	219.89 ± 13.46 cdefgh
WIR453	121.00 ± 6.26 abc	Everglades 41	149.56 ± 8.79 def	WIR453	178.67 ± 12.39 bcde	WIR276	215.67 ± 7.45 defghi
WIR360	117.00 ± 7.24 abc	WIR360	149.44 ± 5.67 def	Everglades 41	174.44 ± 6.44 cde	WIR119	210.44 ± 5.06 efghi
PI468075	116.00 ± 18.56 abc	PI365441	143.50 ± 8.46 def	Local	171.33 ± 10.15 cde	WIR214	197.89 ± 8.12 fghi
EF-1	112.33 ± 4.26 bc	PI468075	140.22 ± 22.48 def	PI468075	166.11 ± 16.43 cde	PI468077	193.67 ± 3.89 ghi
PI468077	110.00 ± 10.02 bc	PI468077	137.00 ± 17.46 def	PI468077	150.83 ± 13.42 de	Local	192.00 ± 17.03 fghi
Cubano	103.33 ± 10.59 bc	Cubano	125.67 ± 22.53 ef	Cubano	148.25 ± 20.64 de	Cubano	148.61 ± 13.92 hi
Kenaf	50.11 ± 4.33 c	Kenaf	66.00 ± 3.98 f	Kenaf	68.22 ± 5.61 e	Kenaf	120.89 ± 9.43 i

^1^ Set 1, Set 2, Set 3, and Set 4 measured on 15 July, 26 July, 12 August, and 5 September. ^2^ Unit = cm. ^3^ Means of ± standard errors followed by different letters within columns are significantly different by Dunn’s test with Benjamini–Hochberg. Non-parametric rank data were used for statistical analysis; however, untransformed data are presented.

**Table 7:** Spearman’s rank correlation among diameter, number of nodes, and height in 23 kenaf germplasms at four growth stages.

	Sets	Number of Nodes	Height
Stem Diameter	Set 1	0.34 **	0.40 ***
	Set 2	0.15 ^NS^	−0.03 ^NS^
	Set 3	0.28 *	0.20 ^NS^
	Set 4	0.65 ***	0.34 **
Number of Nodes	Set 1	1	0.57 ***
	Set 2	1	0.62 ***
	Set 3	1	0.69 ***
	Set 4	1	0.67 ***

Set 1, Set 2, Set 3, and Set 4 measured on 15 July, 26 July, 12 August, and 5 September. * Significant at the 0.05, ** Significant at the 0.01, and *** Significant at the 0.001 probability level. ^NS^, nonsignificant at *p* < 0.05.

## Data Availability

Not applicable.
